# Presence and shedding dynamics of Atypical Porcine Pestivirus (APPV) in semen of breeding boars: implications for virus transmission in pig production

**DOI:** 10.1186/s12917-026-05514-8

**Published:** 2026-04-29

**Authors:** Anna Bergfeldt, Frida H Aae, Mette Myrmel, Birgit Ranheim, Maria Stokstad, Randi Sørby

**Affiliations:** 1 Department of Preclinical Sciences and Pathology, Faculty of Veterinary Medicine, Norwegian University of Life Sciences, PO box 5003, Ås, 1432 Norway; 2 Department of Production Animal Clinical Sciences, Faculty of Veterinary Medicine, Norwegian University of Life Sciences, PO box 5003, Ås, 1432 Norway; 3 Virology Unit, Faculty of Veterinary Medicine, Norwegian University of Life Sciences, PO box 5003, Ås, 1432 Norway; 4 Norwegian Meat and Poultry Research Centre, Animalia AS, PO box 396-Økern, Oslo, 0513 Norway

**Keywords:** Atypical porcine pestivirus, Congenital tremor type A-II, Semen shedding, Virus transmission, Breeding boars

## Abstract

**Background:**

Atypical porcine pestivirus (APPV), genus *Pestivirus*, is the causative agent of congenital tremor type A-II, a neurological disease in newborn piglets that is characterized by tremors. Although the virus can spread horizontally, clinical disease is observed only after *in utero* infection. While venereal transmission has been proposed as a potential route, the pathogenesis and transmission dynamics of APPV remain incompletely understood. In this study, we aimed to investigate the presence of APPV in semen from Norwegian breeding boars, its temporal shedding patterns, and the potential impact of APPV on semen producing organs.

**Results:**

An initial screening of 110 breeding boars revealed that 50.9% (56/110) were APPV-positive in semen, as determined by reverse transcription quantitative PCR (RT-qPCR). The virus load in semen was highly variable, with concentrations up to 4.0 × 10^6 genomic copies (GC)/mL. Extended sampling of a smaller subset of APPV-positive boars (*n* = 19) revealed that virus shedding in semen typically persisted for less than 3 months, although intermittent shedding for up to 7 months was observed in one boar. At slaughter, the virus was detected in reproductive organs and accessory glands - even in boars with negative semen and serum samples - but it was not associated with histopathological lesions.

**Conclusions:**

As artificial insemination (AI) is widely used in all tiers of pig production, APPV-positive semen from breeding boars may represent an important source of virus transmission. To assess the viability of APPV in AI semen and clarify its potential role in transmission, further research, such as virus isolation for infectivity studies and experimental infections in naïve sows, are required.

**Supplementary Information:**

The online version contains supplementary material available at 10.1186/s12917-026-05514-8.

## Background

Congenital tremor (CT) type A-II in piglets has been known for over a century, but its causative agent, atypical porcine pestivirus (APPV), was first identified in 2016 [1, 2]. APPV is an RNA virus in the *Pestivirus* genus, which also includes important livestock viruses like bovine viral diarrhoea virus (BVDV), classical swine fever virus (CSFV), and border disease virus (BDV). These viruses can infect fetuses *in utero*, leading to various outcomes depending on the timing of infection, such as neurological disease, malformations, or abortion. Horizontal transmission of APPV is subclinical, while vertical transmission with APPV may lead to birth of piglets with activity-induced tremors, impaired movement and feeding, and increased pre-weaning mortality [1–4].

APPV is endemic in many countries globally, causing outbreaks with variable morbidity in litters (< 10–100%) [2, 5]. Following viremia, APPV can be detected in secretions like saliva, urine, and semen, as well as in blood and feces [2, 4, 6]. Although the standard duration of viremia is not well-defined, it appears to last longer following vertical transmission compared to horizontal transmission, with detection up to 7.5 months of age [2–4, 7]. Horizontal infection may induce long-lasting protective immunity, as it is associated with sustained presence of high levels of APPV neutralizing antibodies [3].

In certain cases, APPV-specific antibodies remain absent in vertically infected, virus shedding pigs, resembling the condition of persistently infected animals [3, 4]. Persistent infection is the result of *in utero* infection during a specific stage of gestation with the pestiviruses BVDV, CSFV and BDV. These offsprings are immune tolerant against the virus, which is shed throughout the animals’ entire lives. This persistent shedding complicates the control and prevention of these diseases.

Factors influencing the spread of APPV between farms remain poorly understood, and the endemic nature of the disease indicates that conventional biosecurity measures often fail to prevent virus introduction. Recently, artificial insemination (AI) has been proposed as a potential transmission route, which aligns with the widespread occurrence of APPV within all levels of the pig production system. Although the transmission of APPV via semen is yet to be confirmed, a 2019 outbreak of CT in Kansas, USA, was considered to result from AI using APPV positive semen [8]. This indicates that APPV infection in semen-producing boars may go undetected.

Despite these concerns, research on APPV presence in AI semen is limited, with variable findings. Gatto et al. (2018) reported a 12.9% detection rate of APPV in semen from four different boar studs in the United States [9]. In contrast, analyses of AI doses from a Swedish boar stud revealed detection rates of 1/24 and 0/100 in samples collected in 2019 and 2021, respectively [10].

Additionally, the duration of virus shedding in semen is important for assessing transmission risk, however the shedding dynamics of APPV remain largely unknown. APPV has been detected in semen or preputial fluid from CT-affected boars up to 6 and 8.5 months of age, but studies on APPV shedding in semen from boars born without signs of CT are lacking [2, 4].

Understanding APPV’s presence in semen involves examining its entry through reproductive gland secretions or crossing of the blood-testis barrier to access the seminiferous tubules. While APPV has been detected in the testes of newborn CT piglets, its presence in mature boar reproductive tissues remains uninvestigated [11, 12].

The extensive use of AI in commercial pig production and the use of chilled, fresh semen contribute to the risk of viable virus transmission. This risk is illustrated by the documented transmission of other porcine viruses through semen. For instance, porcine reproductive and respiratory syndrome virus (PRRSV) and CSFV in semen have been confirmed as infection sources experimentally [13–15].

In Norway, a single breeding boar typically contributes to about 650 litters per year [16]. Given the high number of doses produced and the wide distribution within the production pyramid, APPV infections in breeding boars could have a significant impact on the risk of virus transmission.

To increase the knowledge on semen as a possible route for APPV transmission, the aims of the present study were to (i) screen for APPV in semen from Norwegian breeding boars, (ii) investigate the temporal variation of APPV shedding in semen from such boars, and (iii) detect APPV in semen-producing organs of breeding boars post mortem and examine any potential pathological effects on the tissue.

## Materials and methods

### Ethics approval and consent to participate

All boars included in the study were owed by a commercial boar stud, and written consent for the use of material was obtained. Semen and blood samples were collected exclusively as part of routine procedures performed by boar-stud personnel. These procedures are considered part of normal animal management and comply with Norwegian animal-welfare legislation. No activities defined as animal experimentation were performed (e.g., FOR-2015-06-18-761, § 2(d)), and therefore no formal ethics approval by the Norwegian Food Safety Authority were required. Importantly, no additional sampling was performed specifically for this study.

Tissue samples from reproductive organs were collected post-mortem from boars culled due to standard management and slaughtered at a commercial abattoir. Following slaughter, carcasses entered the normal food-production chain. Stunning and killing followed standard abattoir protocols in accordance with relevant EU animal-welfare legislation and were carried out under the supervision of official veterinarians.

### Study design

**Fig. 1:**
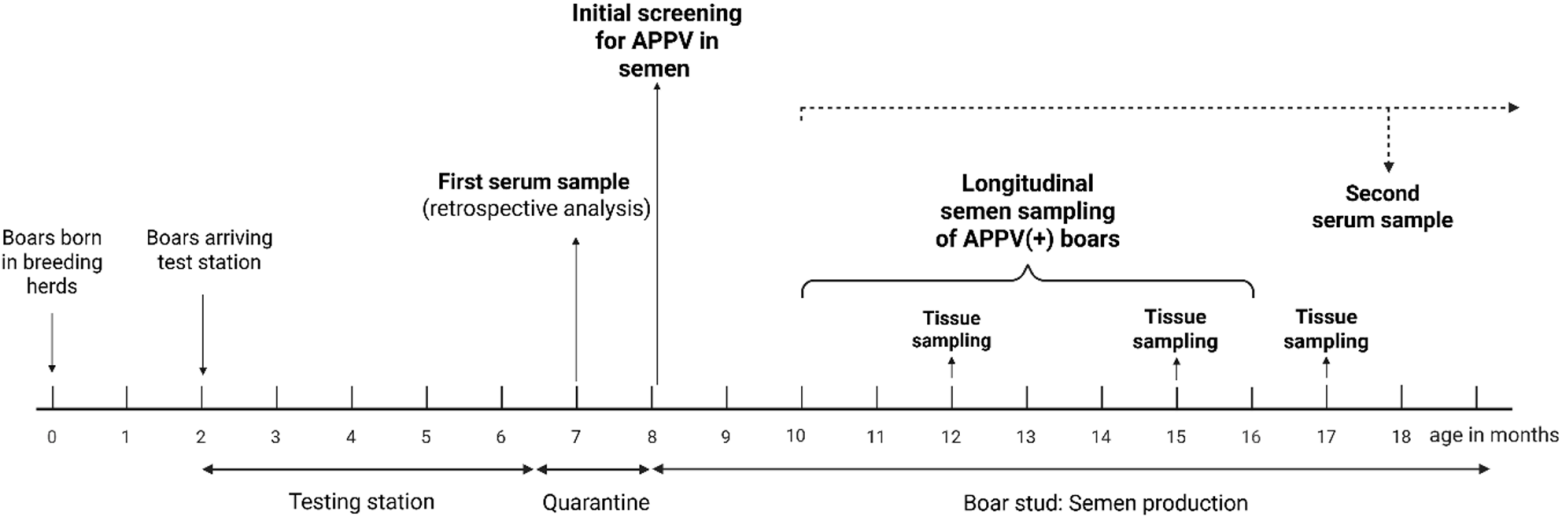
Timeline showing the housing of breeding boars and the timing of sample collection

The study was conducted as a combined cross-sectional and cohort study, where 110 boars from the boar stud were initially screened for presence of APPV in semen, followed by longitudinal testing of semen samples from a stratified group. Serum samples from a selection of the boars were also analysed for APPV at two time points, and tissue samples from reproductive organs, from a subgroup, were investigated at slaughter. A timeline for sampling and housing of the breeding boars is provided in Fig. 1.

### Boar stud

The Norwegian boar stud supplied semen for the majority of domestic commercial herds. The boars were born in breeding herds throughout the country and arrived at a test station at approximately eight weeks of age (Fig. 1). Detailed health records, including mandatory reporting of congenital tremor, were kept by the breeding herds. None of the boars arriving the test station during the study period had a history of CT.

Boars from different herds were mixed at the test station, where they stayed for approximately 10–12 weeks. Boars that qualified for semen production moved to a quarantine station for six weeks before being transported to the boar stud for semen production. Around 300 breeding boars were continuously housed at the boar stud, and they were typically in production for 1–2 years, with new groups arriving every 6 weeks.

### Included animals and sampling flow

For the cross-sectional screening, 110 individual boars were sampled from three consecutive arrival groups at the boar stud (Table 1). These groups were not defined by the study design but reflect the routine management of the stud, and each arrival group consisted of a mixture of boars originating from multiple breeding herds. The boars were approximately eight months old at the time of the screening.

A stratified group of 19 APPV-positive boars from the initial semen screening was selected for longitudinal sampling. The selected boars constituted a group with a broad range of APPV levels, although individuals with very low viral loads were mostly excluded from follow-up sampling. The boars were sampled at two to four time points, approximately every four weeks, over a period of about two months. The interval between the initial screening and the first longitudinal sampling varied from two to six months (Table 1). This variation reflects the routine flow at the boar stud: semen samples from the three consecutive arrival groups were analyzed simultaneously before selecting individuals for follow-up. Consequently, more time had elapsed for boars from the earliest arrival group than for those from the most recent group.

**Table 1:** Group origin and number of boars in the initial and longitudinal APPV semen studies

Group	Boars in initial screening	Boars in longitudinal study	Time between initial screening and first longitudinal sample (months)
A	35	5	6
B	34	4	5
C	41	10	2
Total:	110	19	

The number of months between the initial screening and first sample in longitudinal study is given

Stored serum samples collected three weeks prior to the semen screening were analysed from a subgroup (*n* = 15) of the 19 APPV-positive boars in the longitudinal study (B1-B19). This analysis also included seven APPV-negative boars. A second serum sample, sampled between two and 17.5 months after the initial screening, was available for APPV analysis from 14 APPV-positive boars and five APPV-negative boars.

For APPV-analysis and pathological examination of reproductive organs and accessory glands, tissue samples were collected at slaughter from six APPV-positive boars included in the longitudinal sampling, and three APPV-negative boars from the initial screening. The boars were slaughtered due to declining semen quality (*n* = 6), low breeding score (*n* = 2), and yielding small litters (*n* = 1). The time of slaughter was four (*n* = 2), seven (*n* = 5) and nine (*n* = 2) months after the initial screening, and one of the three APPV-negative boars was included at each time point (Fig. 1).

**Fig. 2:**
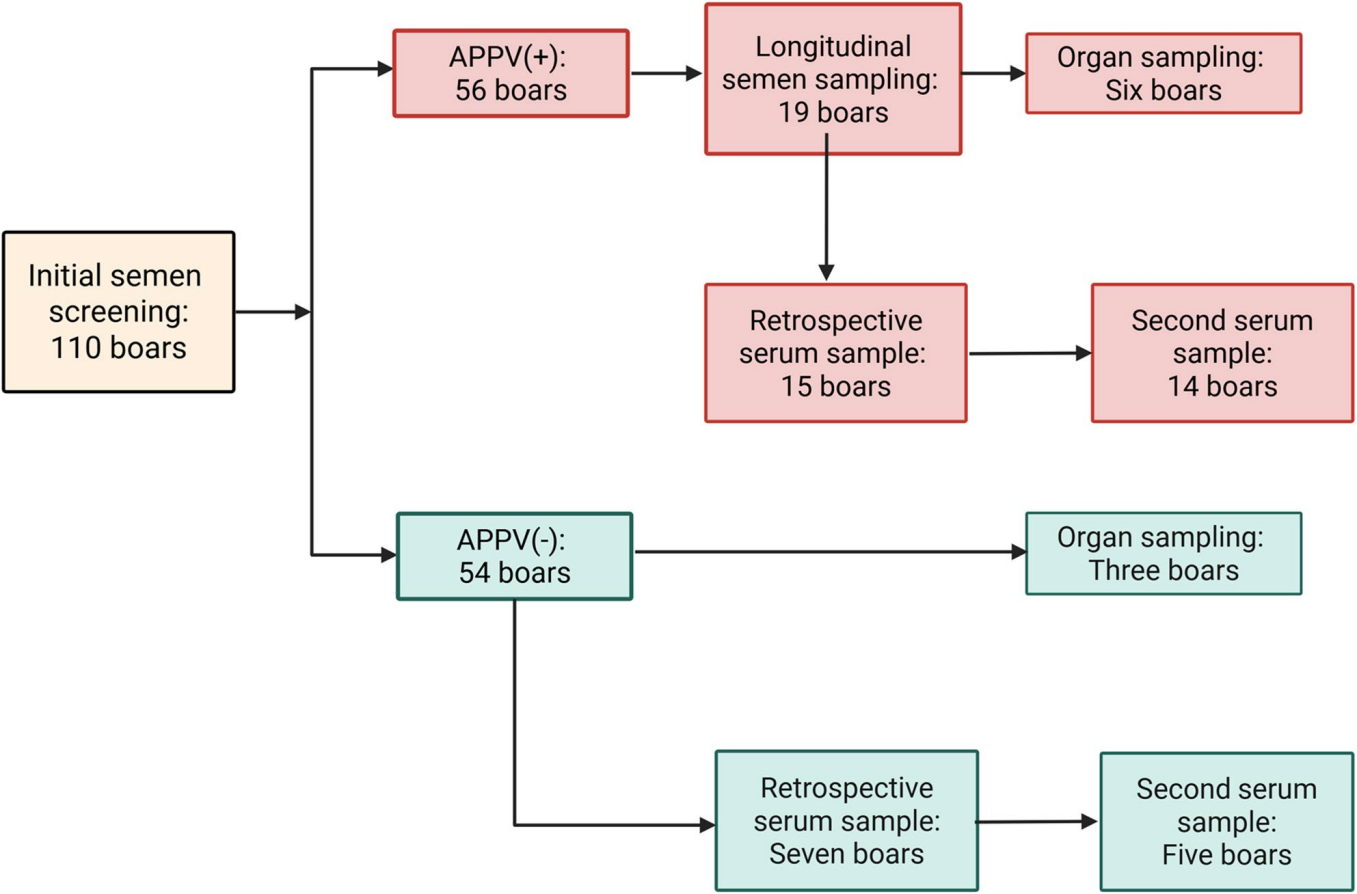
Flow diagram of included animals and sampling

An overview of all samples is found in Fig. 2.

### Biomaterial (semen, serum and tissue)

Semen and serum samples were provided by the boar stud. Semen samples were collected and immediately frozen at -20 °C at the boar stud facilities. Samples were transported on dry ice to the research facilities and transferred to -80 °C for long-term storage. All samples underwent a single freeze-thaw cycle prior to RNA extraction, as RT-qPCR was performed on the same day from the same thawed aliquot. At the boar stud, the sperm-rich fraction was collected by trained personnel, and no additional fractionation of the samples was performed.

Boars included in the longitudinal sampling were assigned the IDs B1-B19. APPV-positive boars not selected for longitudinal sampling were assigned IDs B20-B56. Three APPV-negative boars were included as negative controls for tissue examination at slaughter and were assigned the IDs B57-B59. No additional control animals were introduced.

Blood samples were collected as part of routine disease surveillance and the resulting serum samples were stored at -80 °C at a diagnostic facility before being transferred to the research facilities for analysis.

At slaughter, tissue samples were collected by study personnel. Samples from the testis, epididymis, prostate, seminal vesicles and bulbourethral glands were obtained approximately one hour after the boars were stunned and bled. The tissue samples were placed in chilled 0.9% saline and transported to the laboratory facility within 30 min. Samples for histology were fixed in 10% neutral buffered formalin, while 40–50 mg tissue were preserved in 1 mL RNA*later* (Thermo Fisher, Massachusetts, United States) for RT-qPCR, kept at room temperature (RT) for 24 h, and stored at -80 °C. Before RNA extraction, 15–19 mg of tissue was subsampled for analysis, and excess RNA*later* was removed by briefly pressing the tissue against the inner wall of the tube.

### Laboratory analyses

#### RNA extraction

RNA extraction was performed using the QIAsymphony SP instrument and corresponding Qiagen kits (Qiagen, Hilden, Germany), following standardized protocols for each sample type.

For semen samples 150 mg of raw semen was thawed, mixed with 750 µL Qiazol lysis reagent (Qiagen, Hilden, Germany), and incubated for 5 min at room temperature (RT). Subsequently, 150 µL of chloroform was added, and the mixture was shaken for 30 s before a 2-min incubation at RT. Phase separation was achieved by centrifugation at 16,000 x g for 20 min at 4 °C in a fixed-angle rotor (Thermo Scientific™ Fresco™ 21 Microcentrifuge). The aqueous phase (400 µL) was then subjected for RNA isolation, yielding a final volume of 50 µL.

For tissue samples, 15–19 mg of thawed tissue was combined with 400 µL of 2 M dithiothreitol (DTT) and a 5 mm steel bead in 2 mL tubes. The samples were homogenized at 20 Hz using a FastPrep^®^-24 homogeniser (MP Biomedicals, Irvine, CA, USA) and subsequently centrifuged at 2,200 x g for 3 min at 4 °C in a fixed-angle rotor. RNA was extracted from 400 µL of the lysate, following the same procedure as for semen samples.

For serum samples, RNA was extracted using the DSP Virus/Pathogen Mini Kit (Qiagen), following the Cellfree200_V7_DPS protocol. The elution volume was set to 60 µL. RNA extracts from tissue and serum were stored at -80*°*C until analysis, while RNA from semen was analysed with RT-qPCR the same day.

#### RT-qPCR for APPV

Isolated RNA from semen, serum and tissue samples was analysed with RT-qPCR targeting the coding sequence for NS5B. The assay employed primers APPV-NS5B-303 F and APPV-NS5B-385R, probe APPV-NS5B-336-FAM, and the RNA UltraSense™ One-Step Quantitative RT-PCR System (Thermo Fischer) as described earlier [17]. To generate a standard curve, a series of 10-fold APPV RNA dilutions were run in triplicate, yielding an amplification efficiency of 104% (E = 1.04, denoting the amplification efficiency increment), a correlation coefficient (R^2) of 0.98 and a slope of -3.23.

The limit of quantification (LoQ) was established at Cq = 35.1, representing the lowest concentration at which the standard curve remained linear. Samples within the quantifiable range (Cq < 35.1) showed consistent duplicate amplification (ΔCq < 1). Amplification above this threshold (Cq > 35.1) was interpreted as detectable but not quantifiable. The limit of detection (LoD) was defined as any reproducible amplification signal within 40 cycles in one or both duplicate reactions. Samples meeting this criterion but falling above the LoQ were classified as non-quantifiable positives.

PCR inhibition by the semen matrix was assessed by spiking APPV-positive material into APPV-negative semen and analyzing 1:1 and 1:10 dilutions. No deviations from expected ΔCq values were observed, which indicated no inhibition. No internal exogenous control was included, which is acknowledged as a methodological limitation. The same positive control was used in all experiments, with its APPV copy number quantified using RT-ddPCR as described below. APPV genomic copies in semen were calculated as N1 = N2 · (1 + E)^ (Cq2 − Cq1), where N2 represents the copy number of the positive control as determined by RT-ddPCR, and Cq1 and Cq2 the cycle threshold values of sample and control, respectively [18]. Genome copies (GC) were expressed per mL of semen. The LoQ (Cq = 35.1) corresponded to 1060 GC/mL semen.

#### RT-ddPCR for APPV

The RT-ddPCR was performed using the QX200 droplet digital system (Bio-Rad, Hercules, California, USA) following the manufacturers protocol. The One-Step RT-ddPCR kit (Bio-Rad) was used with a final concentration of 450 nM of each primer and 250nM of the probe (the same primers and probe as in the RT-qPCR assay), and 2 µL RNA in a total volume of 20 µL. Sample emulsification was carried out with oil (Bio-Rad) in the QX200 droplet generator, and 20 µL of the emulsified sample was transferred to a 96-well plate for RT-PCR on a C1000 Touch Thermal Cycler (Bio-Rad). Temperature settings for reverse transcription and annealing were optimized across a range of 40–50 °C and 50–60 °C, respectively. The final thermal cycling settings were as follows: reverse transcription at 42 °C for 1 h, followed by PCR at 95 °C for 10 min, 40 cycles of 95 °C for 30 s and 52.7 °C for 60 s, and a final step at 98 °C for 10 min. Droplet fluorescence was analysed using QX200 Droplet Reader (Bio-Rad), with quantification performed only for samples containing a minimum of 10,000 accepted droplets. APPV genomic copies (GC) were expressed as GC/µL of RNA analyzed, using QuantaSoft Software version 1.7.4 (Bio-Rad).

#### Histological evaluations

Formalin-fixed tissue samples were dehydrated, embedded in paraffin wax, and sectioned in 4 μm slices. The sections were mounted on glass slides and stained with haematoxylin and eosin following standardized protocols. Histological examination was performed using digitally scanned slices (Philips, IntelliSite Ultra-fast Scanner).

### Statistical analyses

Descriptive statistics were calculated using Jamovi software (version 2.3.18). The 95% confidence interval for the prevalence of positive boars was calculated using the Wald method. Further statistical analyses were performed in STATA/SE 17.0 (StataCorp, College Station, TX, USA). To evaluate the duration of viral shedding, a survival analysis was performed. For each boar, the number of days from the initial screening until the last positive sample was calculated. Kaplan-Meier curves were produced and stratified by quartiles of the initial viral load (GC/mL). A Cox proportional hazard model was used to assess whether initial viral load predicted time to negativity. Proportional hazards assumptions were evaluated using Schoenfeld residuals, with no evidence of violation.

Results were considered statistically significant at a p-value < 0.05.

## Results

### Initial screening of APPV in semen

APPV was detected in semen from 50.9% of the screened breeding boars (56/110, 95% CI: 0.42–0.60). Detection rates were similar across the three arrival groups: 49% (17/35) in group A, 50% (17/34) in group B, and 54% (22/41) in group C (Supplementary Table S1).

Three semen samples contained APPV concentrations exceeding 1.0 × 10^6 GC/mL, with the highest level reaching 4.0 × 10^6 GC/mL. An additional four samples had concentrations above 1 × 10^5 GC/mL. Fourteen samples contained 1.0 × 10^4 to 1.0 × 10^5 GC/mL, while 35 samples (64%) had concentrations below 1.0 × 10^4 GC/mL. Among these 35 samples, 20 tested positive with non-quantifiable APPV-levels (< 1060 GC/mL). A summary of the viral load results from the screening is given in Supplementary Table S1.

### Longitudinal sampling for APPV in semen

**Fig. 3:**
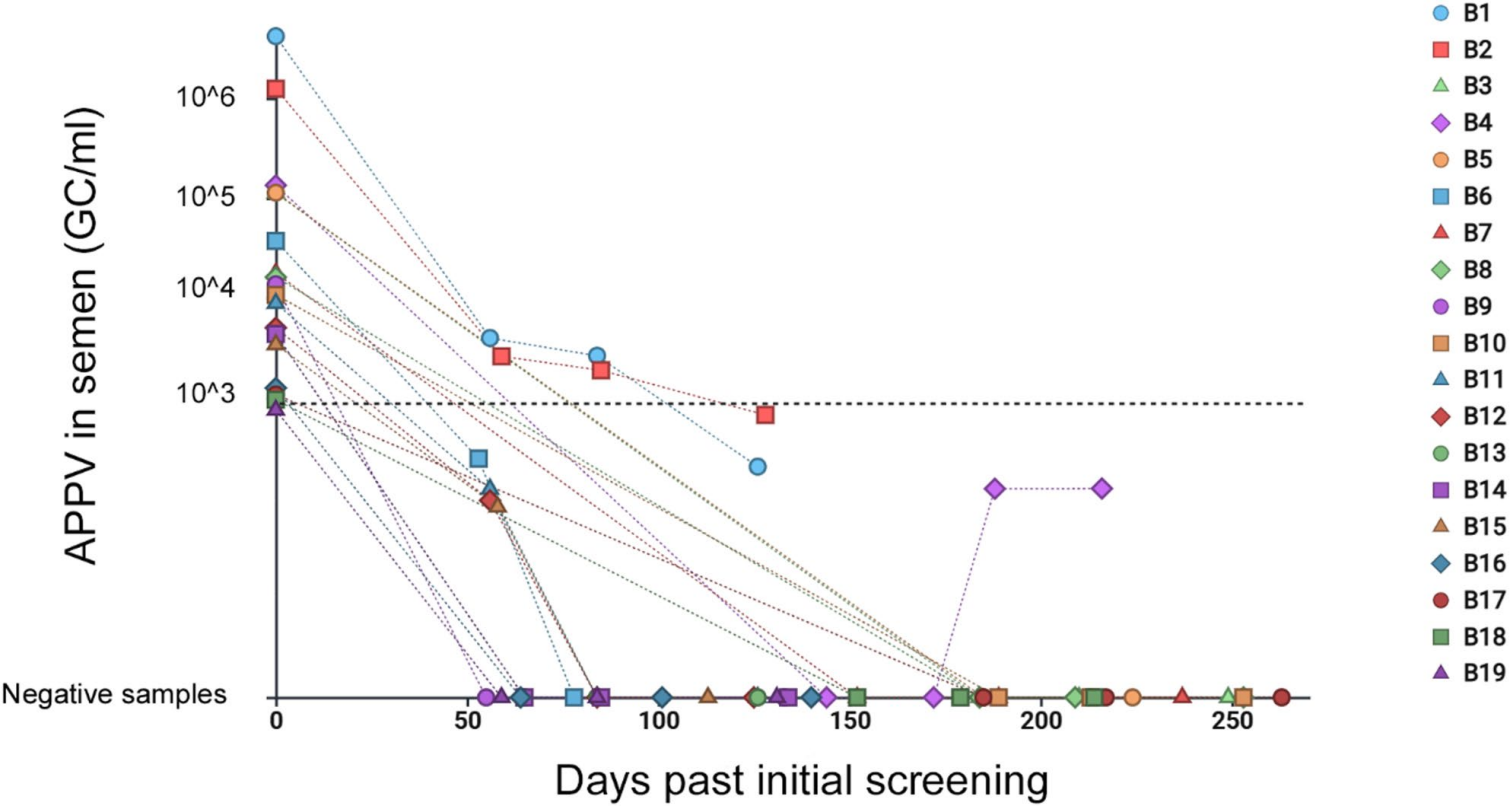
Development of APPV load in semen, from initial screening through longitudinal study. APPV load is presented as genomic copies (GC)/mL. Each point represents an individual semen sample, and colors indicate different boars (B1-B19). Some data points overlap due to similar values. The horizontal dashed line represents the limit of quantification (LoQ = 1.06 × 10^3 GC/mL; Cq 35.1). Symbols plotted below the LoQ line represent non-quantifiable positives, whereas points at the x-axis represent true negative samples

The 19 boars included for longitudinal sampling (B1-B19) exhibited considerable variation in APPV concentrations in semen at the initial screening (Fig. 3), and their shedding patterns over time reflected this variability. Duration of confirmed APPV-shedding from the first semen screening is shown in Table 2. At two months after the initial screening, 60% still shed the virus in semen. This proportion declined with time, with no detection after five months in most individuals. One boar exhibited intermittent shedding, testing positive at six and seven months after the initial screening despite having apparently cleared the virus in previous samples.

**Table 2:** Monthly distribution of boars with APPV-positive semen after the initial screening

Months after initial screening	No. positive/total
0	19/19 (100%)
2	6/10 (60%)
3	2/10 (20%)
4	2/10 (20%)
5	0/4 (0%)
6	1/8 (12.5%)
7	1/8 (12.5%)
8	0/4 (0%)
9	0/1 (0%)

The number of boars tested at each time point is given. The number tested per month varied because the sampling of the three arrival groups overlapped in time, and the number of individuals from each group available for testing differed between months (A = 5, B = 4, and C = 10)

The number of boars tested at each time point is given. The number tested per month varied because the sampling of the three arrival groups overlapped in time, and the number of individuals from each group available for testing differed between months (A = 5, B = 4, and C = 10).

Among the six boars that tested positive two months after the screening, two had virus levels exceeding 10^3 GC/mL, and their APPV concentration remained stable for another month (Fig. 3). These two boars had the highest APPV concentration at the initial screening and they also tested positive at their last sampling, four months past the initial screening, with virus levels below the level of quantification. The other four boars had non-quantifiable levels of APPV after two months, and none of them tested positive after three months. One boar tested positive with non-quantifiable levels of APPV at six and seven months, following two previous samplings with negative results.

**Fig. 4:**
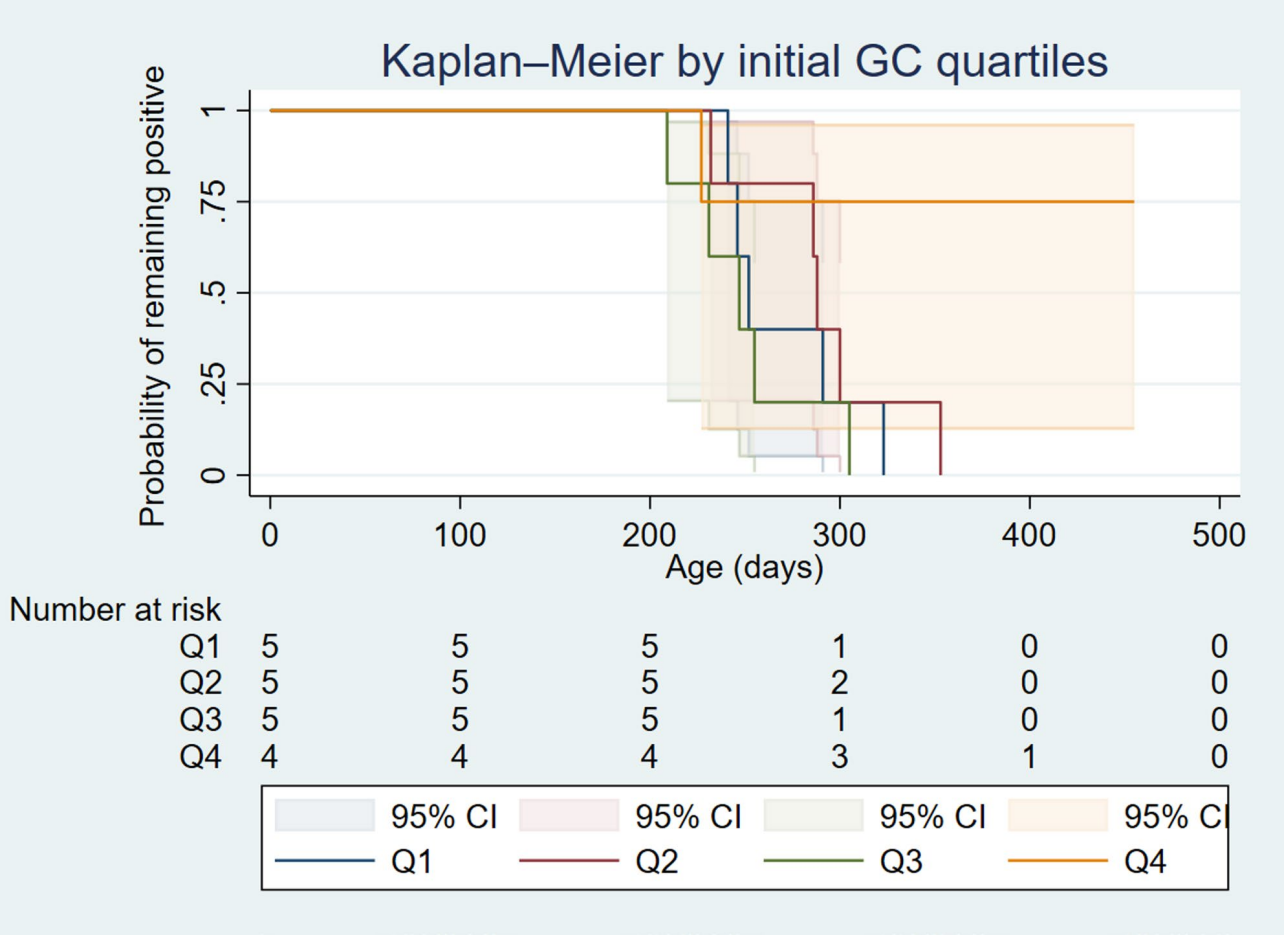
Kaplan-Meier curves for time to negativity by quartiles of initial viral load (genomic copies (GC) /mL). The curves show the probability of remaining virus-positive over time for all 19 boars included in the longitudinal sampling. Boars were divided into quartiles (Q) based on their initial viral load (Q1: lowest; Q2: low-medium; Q3: medium-high; Q4: highest). Shaded areas represent 95% confidence intervals. “Numbers at risk” indicate how many animals remain positive at each time point

The Kaplan-Meier curve showed that boars with higher viral loads tended to remain positive for longer, although the confidence intervals overlapped (Fig. 4). In the Cox-model, initial viral load was not significantly associated with time to negativity (HR = 0.99; 95% CI = 0.99-1.0; *P* = 0.21).

### APPV in serum

All serum samples were APPV negative at both time points.

### APPV in reproductive organs and accessory glands

Four out of six APPV-positive boars had detectable, but non-quantifiable, levels of APPV in one or more reproductive organs at the time of slaughter (Table 3). Viral levels were low (Cq > 35.1; < LoQ), with the highest detected in the epididymis of the boar sampled four months after the initial screening. At seven months past the initial screening, three of four boars had APPV RNA detectable in one or two semen-producing organs, whereas the boar sampled nine months after screening tested negative in all organs. All three APPV-negative control boars (B57-B59) were negative in every reproductive tissue examined.

Four of the boars were included in the longitudinal sampling and had blood and semen collected the day before slaughter (Table 3; Fig. 3). The boar with APPV detected in the testicle (B4), had also tested positive in semen for two months despite two preceding negative samples. The other three boars with detectable APPV in reproductive tissues (B5, B8, B17) had been semen-negative for at least three consecutive months prior to slaughter. None of the boars were APPV positive in serum at the time of sampling.

**Table 3:** APPV RT-qPCR results from breeding boars

Boar ID	Semen APPV (initial)	Months after initial	Serum	Semen	Reproductive tissue APPV (Cq > 35.1; < LoQ)*
B17	+	4	-	-	Epididymis: Cq 35.3
B57	-	4	-	n/a**	-
B5	+	7	-	-	Seminal vesicles: Cq 38.4
B4	+	7	-	+***	Testicle: Cq 39.0
B18	+	7	n/a**	n/a**	-
B8	+	7	-	-	Prostate: Cq 36.6 Seminal vesicles: Cq 37.6
B58	-	7	n/a**	n/a**	-
B7	+	9	n/a**	n/a**	-
B59	-	9	n/a**	n/a**	-

Concurrent sampling was performed from the reproductive tract, serum and semen. Reproductive organs were sampled at slaughter (the number of months after initial semen screening is shown), while blood and semen were collected the day before slaughter. B57-B59 are APPV-negative boars identified during the initial semen screening and included as negative controls for tissue examination

*Cq-values in reproductive tissues were below the limit of quantification (Cq > 35.1), corresponding to < 1060 GC/mL. **n/a: Not available. *** In boar B4, APPV RNA was detected in semen at the sampling before slaughter (Ct 38.1), below the quantification limit

### Gross and histopathological findings in reproductive organs

No gross morphological changes were observed in any organs, and most boars (6/9) showed no histopathological changes (Supplementary Fig. 1). Among the three boars with abnormalities, two (B57 and B59) had tested APPV-negative at the initial semen screening, while one (B8) tested positive.

Boar B57, exhibited multifocal, mild to moderate, chronic-active inflammation in the accessory glands (prostate, seminal vesicles, bulbourethral glands) (Supplementary Fig. 2a). This boar tested APPV negative at the initial semen screening and was slaughtered due to reduced semen quality.

Multifocal deposits of mineralized concretions within the prostate glands were observed in boars B8 and B59. Boar B59 also exhibited varying degrees of epithelial degeneration near the concretions (Supplementary Fig. 2b). This boar was slaughtered due to small litter size and tested APPV negative at the initial semen screening. Boar B8, which tested APPV positive initially, had low levels of APPV in the prostate and seminal glands seven months later, and was slaughtered due to reduced semen quality.

## Discussion

The aim of the study was to investigate the presence of APPV in raw semen among Norwegian breeding boars, evaluate the fluctuation of virus shedding over time, and assess the presence of APPV in reproductive organs and any possible pathological effects on these tissues.

In our initial screening, more than 50% of the boars tested positive for APPV in semen, a result that contrasts with lower prevalence rates reported in previous studies [9, 10]. The lower detection rates in the Swedish study may partly be due to analyzing ready-to use AI doses, in contrast to the undiluted raw semen examined in our study and that of Gatto et al. (2018) [9, 10]. The addition of a large volume of extender to AI doses significantly dilutes the viral load originating from raw APPV-positive semen. Furthermore, commercial AI doses may originate from either individual boars or be pooled from a mixture of boars, which may increase the probability of APPV presence but reduce the viral load per dose.

Boar age may also influence the detection rate of APPV in AI semen, as breeding boars can remain in production for several years and are unlikely to experience reinfections with APPV [3]. In our longitudinal sampling, we observed a progressive decline in APPV prevalence in boars over time following the initial screening. Since all boars in our study were approximately 8 months old and at the onset of their semen production period, these findings suggest that APPV detection may be affected by age-dependent factors. Previous studies did not specify the age of the included boars, but a higher average age among boars could help explain the lower prevalence rates reported in those studies.

APPV-positive semen may result from either transient horizontal infection or infection acquired *in utero*. In our study, vertical transmission appeared unlikely, given that the boars had no history of congenital tremor, and all tested boars were serum PCR-negative at two time points surrounding semen positivity, which is inconsistent with classical pestiviral persistent infection (typically characterized by sustained viremia and absent seroconversion). While negative serum PCR cannot fully exclude *in utero* infection, as APPV RNA may persist longer in feces or preputial fluid after vertical exposure, current evidence indicates that serology alone would not resolve the infection route [2, 4]. Congenitally infected pigs show highly variable antibody responses, and truly antibody-negative infections appear to be uncommon [3, 7]. Previous longitudinal studies indicate that horizontally infected pigs typically clear viremia within approximately two months and then seroconvert, whereas pigs infected in utero tend to remain PCR-positive for longer and display heterogenous serological patterns [3, 7]. Taken together, and despite the lack of serology as an acknowledged limitation, the repeated serum-negative status of the boars, together with no history of clinical signs, supports transient horizontal infection rather than classical pestiviral persistent infection.

The combination of low-level detection of APPV in semen, intermittent reappearance after periods of negativity, and the absence of systemic viremia is compatible with localized, low-grade viral persistence in immune-privileged compartments of the male reproductive tract. A comparable phenomenon is described for BVDV, where seropositive, non-viremic post-pubertal bulls can sustain testicular or epididymal infection and shed virus in semen for extended periods despite systemic clearance [19, 20]. Although the phenomenon is rarely observed, this mechanism offers a potential explanation for the extended and intermittent APPV shedding in semen observed in one boar, although confirmation regarding APPV requires further investigation.

Determining the precise timing of infection is difficult due to limitations in our study design. It is most probable that the boars acquired the infection during the testing period, but infection during the quarantine period cannot be ruled out. The detection rates of APPV-positive boars may reflect either a constant presence of APPV in young Norwegian breeding boars or a temporary, time-dependent spread. In the first scenario, virus circulation is sustained by regularly introducing susceptible individuals to the test station. Alternatively, our findings may result from a subclinical, horizontal infection at the test station. This could have occurred before the initial semen sampling, following the introduction of a virus shedding boar. As CT outbreaks occur at all levels of the breeding pyramid, this situation might arise from a reporting error or unnoticed low-morbidity CT in one of the breeding herds. In this scenario, APPV detection rates in semen would be influenced by the sampling time in addition to the age of sampled boars. This time-dependent variation is supported by the findings of Gatto et al., whose study showed that APPV-positive semen samples varied between 6.8 and 34.9% across boar studs, and from 0.0 to 23.0% within a single boar stud over a 1.5-year period [9]. Regardless of the transmission dynamics of APPV at the boar stud, the subclinical presentation following horizontal infection allows the virus to spread undetected [1–3].

Subclinical infections and the relatively brief duration of viremia following horizontal transmission complicate screening for APPV-positive boars [3, 7]. In our study, boars that later tested APPV-positive in semen were serum-negative three weeks earlier, indicating that routine serum testing before entry to the stud may fail to identify shedding animals. Although semen testing would minimize false negatives, it is not feasible for young boars and those not trained to ejaculate. Preputial fluid or swabs could serve as an alternative, but their diagnostic reliability remains uncertain, as correlations between preputial and semen APPV detection have not been systematically evaluated [2, 9]. At present, it remains premature to propose defined screening strategies, given that the infectivity of APPV in semen, the minimum infectious dose, and the efficiency of venereal transmission have not yet been established. Nevertheless, our findings underline several aspects that warrant evaluation in future studies, including the potential value of preputial sampling in young boars, the effects of extender dilution and pooling practices on diagnostic sensitivity, and the challenge posed by intermittent shedding for clearance testing. Once the transmissibility of APPV via AI semen is experimentally confirmed and the infectious dose clarified, an evidence-based discussion of routine screening approaches will be possible.

The presence of APPV in AI semen represents a potential transmission route. Infection could occur directly in the gilt or sow, leading to viremia, or indirectly through onward transmission within the herd. If infected animals infected animals subsequently expose other pregnant, susceptible sows during a sensitive gestational window, in utero infection of fetuses may occur. Although no experimental studies have investigated APPV transmissibility via AI, other pestiviruses, such as BVDV and BDV, are known to cause seroconversion through insemination [21–23]. Previous inoculation trials have shown that pregnant sows and weaner pigs can be infected using serum or pooled tissue homogenates from APPV-positive pigs via various routes [2]. The total APPV loads in these inocula were comparable to the highest loads observed in raw semen in the present study, but the minimum infectious dose remains unknown [2]. Thus, a lower dose might also be infective. Additionally, while an orofecal transmission is believed to be the dominant route for APPV, the conditions required for venereal transmission remain unclear, including virus viability in semen extenders during cold storage and the ability of the virus to overcome mucosal and immunological barriers in the female reproductive tract [2, 4, 24].

We observed decreasing levels of APPV in semen for up to 216 days, a duration considerably longer than the observed shedding periods of other viruses in boar semen [25, 26]. Although APPV levels were low in several samples, an extended shedding period increases the likelihood of transmission, as the virus may be present in more AI doses. Notably, the reappearance of APPV in semen from one boar after two months of negative results indicates intermittent virus shedding may occur. While reinfection in this boar cannot be excluded, the sustained high levels of APPV neutralizing antibodies in horizontally infected pigs suggest that reinfection is unlikely [3]. Intermittent shedding, however, complicates surveillance and control efforts, as boars that test negative may later resume shedding.

In the longitudinal cohort, boars with higher initial viral loads showed a tendency toward extended shedding, although this was not statistically significant. The Cox model yielded a hazard ratio close to 1, indicating that initial viral load in our study sample had a minimal effect on time to negativity. The small sample size and the number of boars still positive at the end of follow-up, likely limited the power to detect such an association.

The detection of APPV in organs involved in germ cell production and maturation, as well as in those producing seminal fluid, suggests multiple entry routes for the virus into semen. The presence of APPV in tissues for up to seven months indicates a persistent infection. However, since our study focused on the reproductive tract, it was not possible to determine whether the infection was confined solely to these organs. Multi-systemic virus persistence has been documented in CT-affected boars for up to 11 months (*n* = 2), with APPV nucleic acid detected in the CNS, large intestine and lymph nodes [11]. In another study by Buckley et al. (2023), horizontally infected pigs (*n* = 4) tested positive in the cerebellum and nasal turbinates despite two previous months with negative serum results [7]. Although male reproductive organs were not examined, these findings indicate that APPV can persist in tissues after systemic clearance following both vertical and horizontal infection, depending on the timing and route of exposure.

Although APPV persisted at low levels for an extended period, the infection in reproductive tissue was not associated with any lesions, suggesting immune evasion. While pestiviruses are known to induce immune tolerance following *in utero* infection, the mechanisms behind prolonged immune evasion after horizontal infection are less well documented [27, 28]. Findings from this study suggests potential mechanisms, including low-level replication and infection of immune-privileged sites such as the testes and epididymis. Other strategies might involve interference with antigen presentation, modulation of immune responses, or replication in macrophages. Further research is needed to confirm the strategies used by APPV to evade immune detection during prolonged infection of the reproductive tract.

This study has several limitations. First, serological data and infectivity testing of semen were not available for inclusion and remain essential to determine the true transmission potential of APPV via AI. Second, although the longitudinal cohort represented a wide range of APPV shedding levels, few organ samples were examined, and these were collected when APPV shedding had ceased or was very low. Because APPV RNA levels in reproductive tissues were below the limit of quantification, cellular-level localization using RNA-ISH or IHC was unlikely to yield interpretable signals and was therefore not attempted. Such localization would nevertheless be valuable, as APPV has been detected in testicular tissue from newborn CT-affected piglets, suggesting that prolonged persistence in reproductive organs may be part of APPV pathogenesis and could contribute to viral transmission [11, 12]. This gap highlights the need for future studies on tissue tropism and persistence in adult males. Together, these factors describe the need for future studies combining virological, serological, and infectivity-based approaches to fully clarify APPV transmission dynamics.

## Conclusion

This study identified a high occurrence of APPV-positive semen among Norwegian breeding boars, potentially representing an important route of transmission to naïve sows through artificial insemination. The sustained and sometimes intermittent shedding of APPV in semen, combined with consistently negative serum PCR results, highlight challenges for surveillance and may allow infected boars to remain undetected.

Horizontal transmission between boars was considered the most likely source of infection. The prolonged detection of APPV in the reproductive tissues, despite the absence of associated lesions, suggests a localized, persistent infection and indicates that APPV may employ mechanisms of immune evasion within immune-privileged compartments of the male reproductive tract. Further research is needed to confirm whether APPV is transmissible via AI semen, to establish the minimum infective dose, and to assess the infectivity and stability of APPV in semen extenders under routine storage conditions.

## Supplementary Information

Supplementary Material 1: Supplementary Table 2 provides a summary of data used for the time of event analysis, including GC/mL in semen at initial screening.

## Data Availability

Data is provided within the manuscript or supplementary information files.
