# Supplementary material for: Presence and shedding dynamics of Atypical Porcine Pestivirus (APPV) in semen of breeding boars: implications for virus transmission in pig production
Source: BMC Vet Res. 2026 Apr 29;22:352. doi: 10.1186/s12917-026-05514-8 (PMC13277022; doi:10.1186/s12917-026-05514-8)
Supplement: Supplementary file 1 — Supplementary Material 1: Supplementary Table 2 provides a summary of data used for the time of event analysis, including GC/mL in semen at initial screening. [file 12917_2026_5514_MOESM1_ESM.docx]

Supplementary data

**Table S1.** Summary of Atypical porcine pestivirus (APPV) viral load in semen from boars positive at the initial screening, expressed as genome copies/mL semen.

| **Boar ID** | **Arrival group** | **APPV load in semen (GC/mL)** |
| --- | --- | --- |
| B1 | C | 4138737 |
| B2 | C | 1240488.8 |
| B3 | A | 1106756.3 |
| B4 | B | 135096.2 |
| B5 | A | 113849.7 |
| B6 | C | 37705.5 |
| B7 | B | 17963.4 |
| B8 | A | 16373.4 |
| B9 | C | 13897.1 |
| B10 | A | 10905.6 |
| B11 | C | 8996 |
| B12 | C | 5122 |
| B13 | C | 3363.2 |
| B14 | C | 4441.3 |
| B15 | B | 3485.3 |
| B16 | C | 1284.6 |
| B17 | A | 1106 |
| B18 | B | <LoQ* |
| B19 | C | <LoQ* |
| B20 | B | 721562.4 |
| B21 | C | 277568.1 |
| B22 | B | 91926.9 |
| B23 | A | 71626.2 |
| B24 | A | 68139.4 |
| B25 | C | 67655.3 |
| B26 | B | 66223.6 |
| B27 | B | 30445.0 |
| B28 | C | 17583.3 |
| B29 | A | 13603.0 |
| B30 | C | 10083.0 |
| B31 | A | 5991.8 |
| B32 | B | 5782.0 |
| B33 | A | 5345.9 |
| B34 | C | 5345.9 |
| B35 | B | 5049.5 |
| B36 | A | 3485.3 |
| B37 | A | 2388.6 |
| B38 | A | 1266.4 |
| B39 | B | <LoQ* |
| B40 | C | <LoQ* |
| B41 | B | <LoQ* |
| B42 | B | <LoQ* |
| B43 | C | <LoQ* |
| B44 | C | <LoQ* |
| B45 | C | <LoQ* |
| B46 | B | <LoQ* |
| B47 | A | <LoQ* |
| B48 | B | <LoQ* |
| B49 | C | <LoQ* |
| B50 | C | <LoQ* |
| B52 | A | <LoQ* |
| B53 | B | <LoQ* |
| B54 | A | <LoQ* |
| B55 | A | <LoQ* |
| B56 | B | <LoQ* |

*LoQ = Limit of quantification (Cq = 35.1; 1060 GC/mL).

| **Boar ID** | **Age at first positive test (days)** | **Age at last positive test (days)** | **No. of virus-positive days** | **Viral load in semen (GC/mL) at initial test** |
| --- | --- | --- | --- | --- |
| B1 | 248 | 374 | 126 | 4138737 |
| B2 | 254 | 382 | 128 | 1240488.8 |
| B3 | 227 | 227 | 1 | 1106756.3 |
| B4* | 239 | 455 | 216 | 135096.2 |
| B5 | 255 | 255 | 1 | 113849.7 |
| B6 | 252 | 305 | 53 | 37705.5 |
| B7 | 247 | 247 | 1 | 17963.4 |
| B8 | 209 | 209 | 1 | 16373.4 |
| B9 | 231 | 231 | 1 | 13897.1 |
| B10 | 286 | 286 | 1 | 10905.6 |
| B11 | 232 | 288 | 56 | 8996 |
| B12 | 244 | 300 | 56 | 5122 |
| B13 | 265 | 323 | 58 | 3363.2 |
| B14 | 232 | 323 | 1 | 4441.3 |
| B15 | 214 | 353 | 139 | 3485.3 |
| B16 | 246 | 246 | 1 | 1284.6 |
| B17 | 241 | 241 | 1 | 1106 |
| B18 | 291 | 291 | 1 | <LoQ** |
| B19 | 252 | 252 | 1 | <LoQ |

**Table S2.** Age, viral load (GC/mL) and duration of APPV-positivity in semen for the 19 boars included in both the initial screening and the longitudinal sampling.

* Boar number B4 had negative semen samples at day 383 and 411 but was positive again at day 427 and 455. ** LoQ = Limit of quantification.


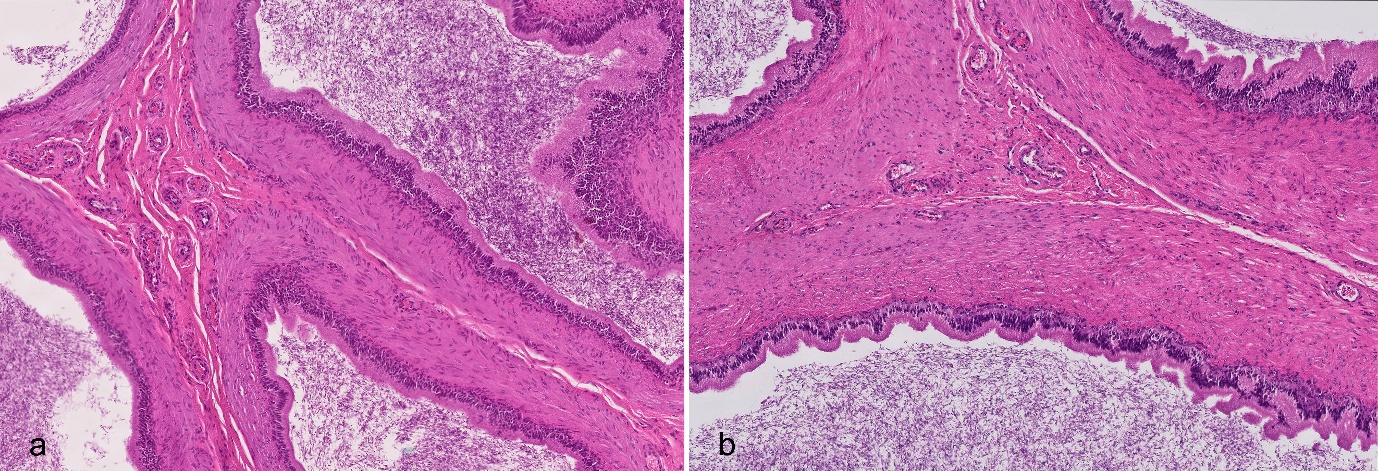


**Figure S1.** Epididymal histology in an APPV‑positive and an APPV‑negative boar. **a)** Representative section from boar B17, which tested APPV‑positive in epididymis by RT‑qPCR before slaughter. **b**) Section from boar B58, which tested APPV negative at the initial screening. No histopathological lesions were observed in either case. H&E-stain. 5x magnification.


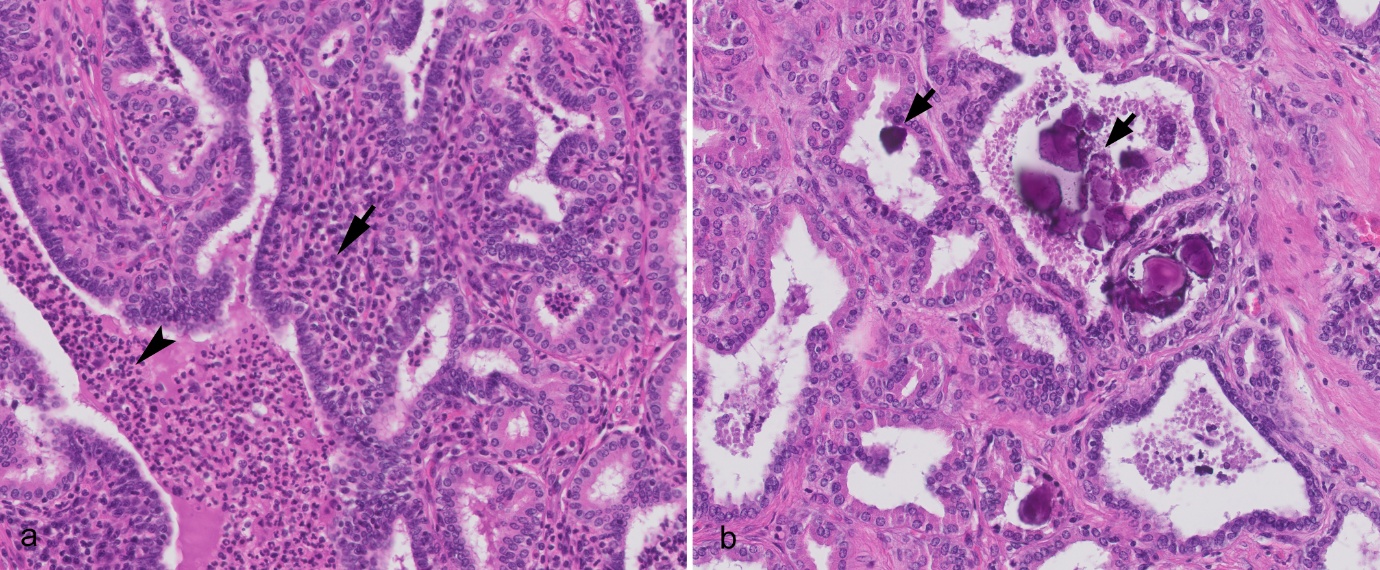


**Figure S2.** Lesions in accessory glands from slaughtered breeding boars. **a)** Seminal vesiculitis, moderate and multifocal, with infiltrates of lymphocytes (arrow) and neutrophil granulocytes (arrowhead) in glands and interstitium. **b)** Multifocal intraglandular and intraductal concretions (arrows) in the prostate gland. H&E-stain. 50x magnification.
